# Auditory symptoms and autistic spectrum disorder: A scoping review and recommendations for future research

**DOI:** 10.1016/j.joto.2022.08.004

**Published:** 2022-08-30

**Authors:** Sara Timms, Sirat Lodhi, Jack Bruce, Emma Stapleton

**Affiliations:** a Department of Otolaryngology, Manchester Royal Infirmary, Manchester, M13 9WL, UK; b The University of Manchester Medical School, Oxford Road, Manchester, M13 9PL, UK

**Keywords:** **KEYWORDS:** Autistic disorder, Audiology, Otolaryngology, Tinnitus, Hyperacusis

## Abstract

**Introduction:**

Auditory symptoms in individuals with Autistic Spectrum Disorder (ASD) are well described within the neurodevelopmental literature, yet there is minimal mention of ASD in Otolaryngology literature. This is surprising considering the potential clinical and diagnostic implications of this link, and the potential for ASD to present to Otolaryngologists in the form of unexplained auditory symptoms. The aims of this literature review were to explore the intersection of auditory symptoms and ASD from the perspective of clinical Otolaryngology, and to outline a clinically focused research agenda based on emerging themes relevant to Otolaryngology.

**Methods:**

We searched Pubmed, Embase, Ovid and Cochrane library for studies until November 2021. Four authors independently reviewed 227 publications identified. 39 were filtered into the final analysis. The PRISMA 2020 guidelines were followed. The heterogeneity of literature meant that a Systematic Review was not feasible. Included studies were therefore classified thematically, forming the basis of the scoping review.

**Results:**

Diagnostic theories for auditory symptoms in ASD include the entire auditory pathway and brain. There is a growing body of literature on auditory symptoms in ASD, suggesting that a primary diagnosis of ASD should be considered in patients presenting with otherwise unexplained auditory symptoms, and indicating a learning need for Otolaryngologists and audiologists, to whom these patients may present.

**Conclusion:**

We recommend a research agenda focusing on multidisciplinary collaboration, stakeholder engagement, responsible clinical screening, and clarification of pathophysiological mechanisms and terminology.

## KEY POINTS

• Atypical responses to the auditory environment in autistic individuals are well documented in neurodevelopmental literature.
• Evidence on the intersection of auditory symptoms and autism is minimal, considering the potential implications of this link.
• Published literature consists of diagnostic theories which include the entire auditory pathway and brain.
• A primary diagnosis of autism should be considered in patients presenting with otherwise unexplained auditory symptoms.
• Future research should incorporate stakeholder engagement and clarification of pathophysiological mechanisms and terminology.

## 1 Introduction

Atypical responses to the auditory environment in individuals with Autistic Spectrum Disorder (ASD) are not a new observation (Rosenhall et al., 1999). There is a body of published work linking auditory symptoms such as tinnitus and decreased sound tolerance (DST) to ASD, largely within the neurodevelopmental literature. Decreased sound tolerance (DST) as a collective term for hyperacusis, misophonia and phonophobia (Jastreboff and Jastreboff, 2001) is used within this manuscript. Several aetiological theories for the link between tinnitus, DST and ASD have been proposed, including processing differences at brainstem (Wilson et al., 2017; Ohmura et al., 2019) and auditory cortex level (Matsuzaki et al., 2012), and abnormal behavioural responses to sound (Lucker, 2013; Stiegler and Davis, 2010). However, evidence regarding the intersection of auditory symptoms and ASD remains surprisingly minimal, considering the potential clinical and diagnostic implications of this link.

A recent meta-analysis (Williams et al., 2021a) concluded that most individuals on the autistic spectrum experience DST at some point in their lives. ASD prevalence studies in child populations (Redfield et al., 2014) suggest that many adults with ASD remain undiagnosed (Brosnan, 2020) and may present in adulthood with medical or neuropsychological complaints. The possibility of an ASD diagnosis in patients presenting to ENT and Audiology services with tinnitus and/or DST could therefore have a significant impact on our clinical practice, highlighting a learning need for professionals in ENT and Audiology, and potentially offering more focused management options to our patients.

Tinnitus and DST present frequently to ENT and Audiology services. Tinnitus has a reported prevalence 5.1%–42.7% (McCormack et al., 2016) in general populations, and hyperacusis 0.2%–17.2% (Ren et al., 2021). These symptoms can have a profound effect on patients’ lives (Nondahl et al., 2007). It is therefore essential that we investigate and manage them optimally.

Tinnitus is defined as a sound in the head or ears that occurs in the absence of any external acoustic source (Baguley et al., 2013). Various diagnostic algorithms aim to identify its origin (Crummer and Hassan, 2004), with bilateral tinnitus of unknown aetiology often being assigned a psychogenic cause (Miura et al., 2017) especially in patients with a history of psychiatric illness. Unilateral tinnitus requires crosssectional imaging (Tunkel et al., 2014) but the incidence of retrocochlear pathology is just 2.7% (Choi et al., 2015), higher in patients with ipsilateral hearing loss. Unilateral tinnitus with no identified aetiology is considered idiopathic and managed with tinnitus retraining therapy (Jastreboff, 2015) based on a neuropsychological model and considered to offer significant help for 80% of patients. Decreased sound tolerance is defined as reduced tolerance to sound, often accompanied by painful sensitivity to ordinary environmental sounds, with perceptual, psychological, and social dimensions (di Stadio et al., 2018). There is currently no widely accepted investigation or treatment pathway, and management strategies focus on behavioural techniques (Potgieter et al., 2020).

The primary aim of this work was to review the literature on ASD and auditory symptoms, exploring the intersection of these themes and identifying evidence for auditory symptoms as a presenting feature of ASD in ENT and Audiology services. A secondary aim was to outline a research agenda based on themes emerging from this work.

**Fig. 1:**
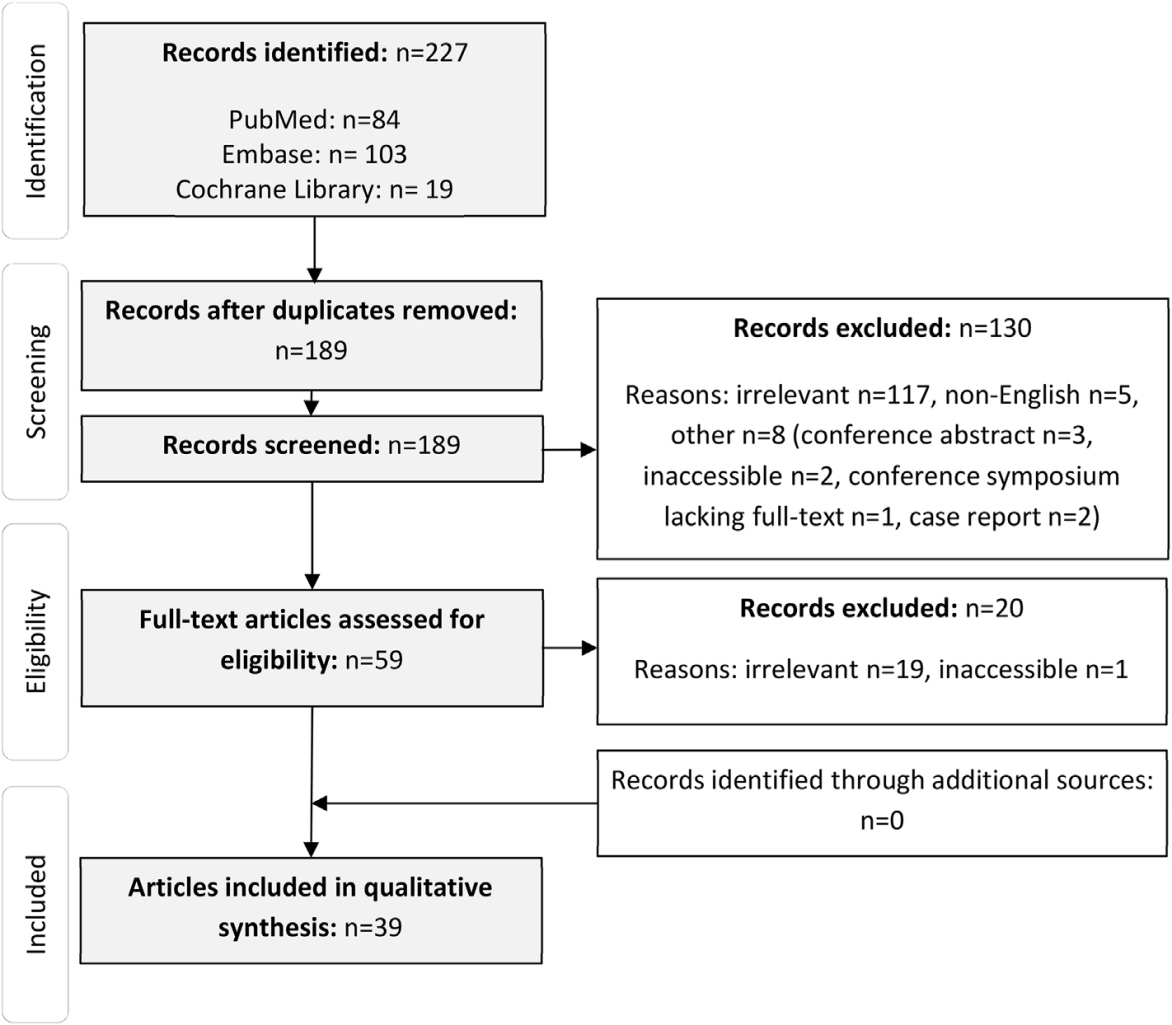
PRISMA flow diagram displaying the systematic search methodology.

**Fig. 2:**
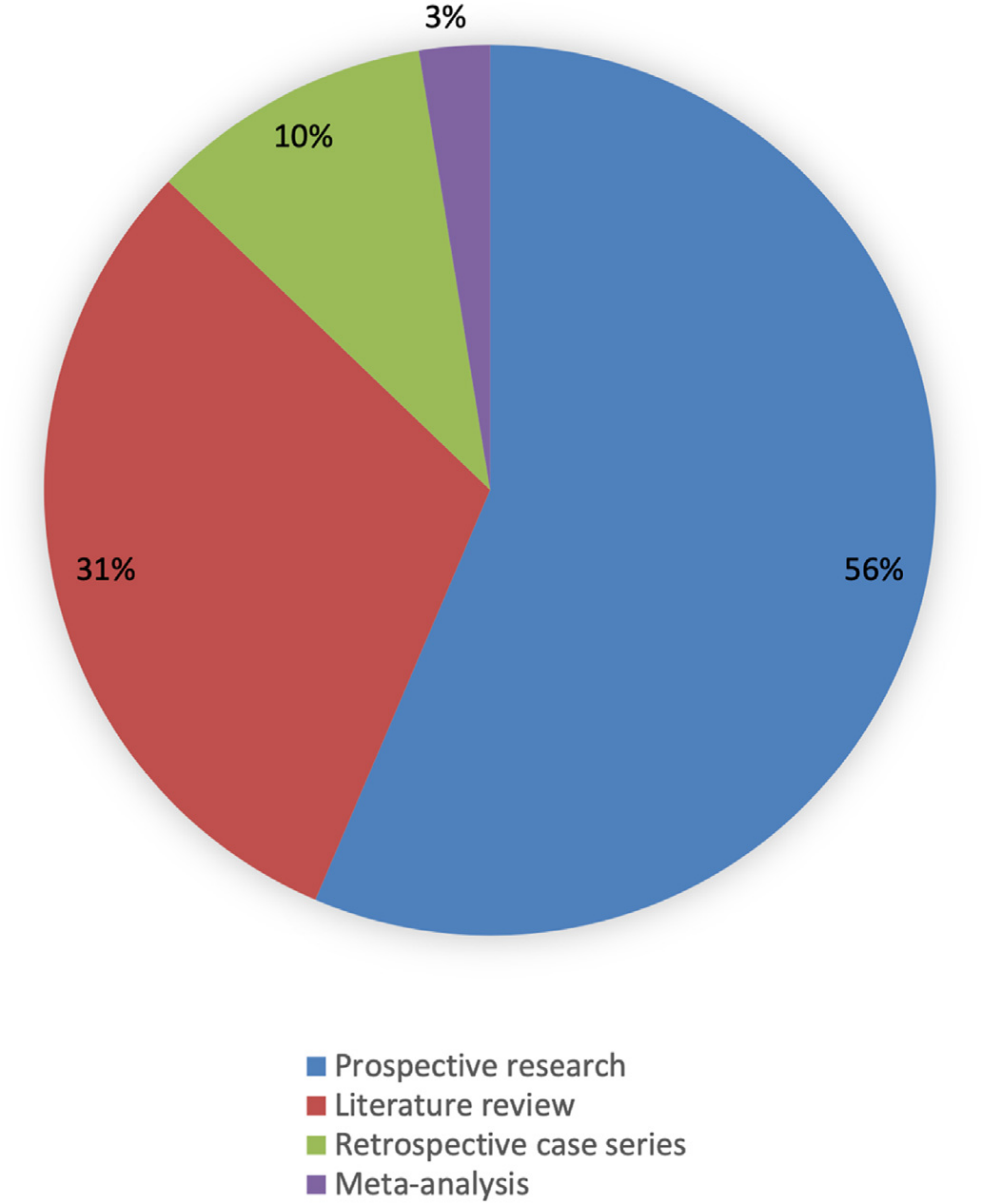
Chart showing literature types included within the analysis.

Our decision to carry out a scoping review followed an initial literature search (Fig. 1) and analysis of current literature (Fig. 2). We discovered that a systematic review would yield little useful information due to literature heterogeneity. Scoping studies can be used to map key concepts underpinning a research area and the evidence available, to identify research gaps in the existing literature (Arksey and O'Malley, 2005). A scoping review was therefore deemed an appropriate model for this work.

## 2 Materials and methods

A methodological framework for scoping studies (Arksey and O'Malley, 2005) was used. 227 relevant publications were identified independently by the authors in November 2021 using PubMed; Embase via OVID; CINAHL Plus via EBSCOhost; and the Cochrane Library (Table 1). Studies were included which explored tinnitus and/or DST in the context of ASD. No restrictions were applied regarding the age of patient populations, or outcomes assessed in the studies.

**Table 1:** Search strategy used to identify relevant publications. Complete search syntax is displayed. Search performed in November 2021.

Database	Search terms
Embase (OVID)	1. Hyperacusis.mp.
	2. Misophonia.mp.
	3. Phonophobia.mp.
	4. Decreased sound tolerance.mp.
	5. DST.mp.
	6. Exp tinnitus/
	7. 1 OR 2 OR 3 OR 4 OR 5 OR 6
	8. exp autism/(includes ASD, autistic disorder, PDD, persuasive developmental disorder(s), kanner syndrome, Asperger syndrome, childhood disintegrative disorder, persuasive developmental disorder not otherwise specified, rett syndrome)
	9. Heller∗.mp.
	10. 8 OR 9
	11. 7 AND 10
PubMed	1. tinnitus [All Fields}
	2. hyperacusis [All Fields]
	3. misophonia [All Fields]
	4. phonophobia [All Fields]
	5. “decreased sound tolerance” [All Fields]
	6. “DST” [Text Word}
	7. 1 or 2 or 3 or 4 or 5 or 6
	8. autism [All Fields]
	9. “autism spectrum disorder”
	10. “autism spectrum condition” [All Fields]
	11. Asperger∗[All Fields]
	12. “high functioning autism”[All Fields]
	13. “childhood disintegrative disorder”[All Fields]
	14. Heller∗ [All Fields]
	15. “atypical autism” [All Fields]
	16. “pervasive developmental disorder”[All Fields]
	17. “PDD-NOS”[All Fields]
	18. 8 or 9 or 10 or 11 or 12 or 3 or 14 or 15 or 16 or 17 19. 7 AND 18
CINAHL Plus via EBSCOhost	1. TX Tinnitus
	2. TX (misophonia or selective sound sensitivity syndrome) OR hyperacusis
	OR phonophobia OR decreased sound tolerance OR DST
	3. 1 OR 2
	4. “(autism or asd or autism spectrum disorder or asperger's or asperger's syndrome or autistic disorder or aspergers) OR autism spectrum condition OR childhood disintegrative disorder OR pervasive developmental disorder OR pdd-nos OR heller∗" 5. 3 AND 4
Cochrane Library	hyperacusis OR misophonia OR phonophobia OR “decreased sound tolerance” OR tinnitus OR “DST” AND
	autism OR “autistic spectrum” OR “persuasive developmental disorder” OR “kanner syndrome” OR
	Asperger∗ OR “childhood disintegrative disorder” OR heller∗ OR “PDD-NOS"

Authors independently screened all study abstracts. 38 duplicates were removed; 117 removed for irrelevant content; five not in the English language and eight for other reasons. 59 studies underwent full-text review by all authors. Twenty were excluded (nineteen irrelevant, one inaccessible). Reference lists and citing papers were screened to identify additional studies. 39 studies were included in the final scoping review. (Fig. 1).

**Fig. 3:**
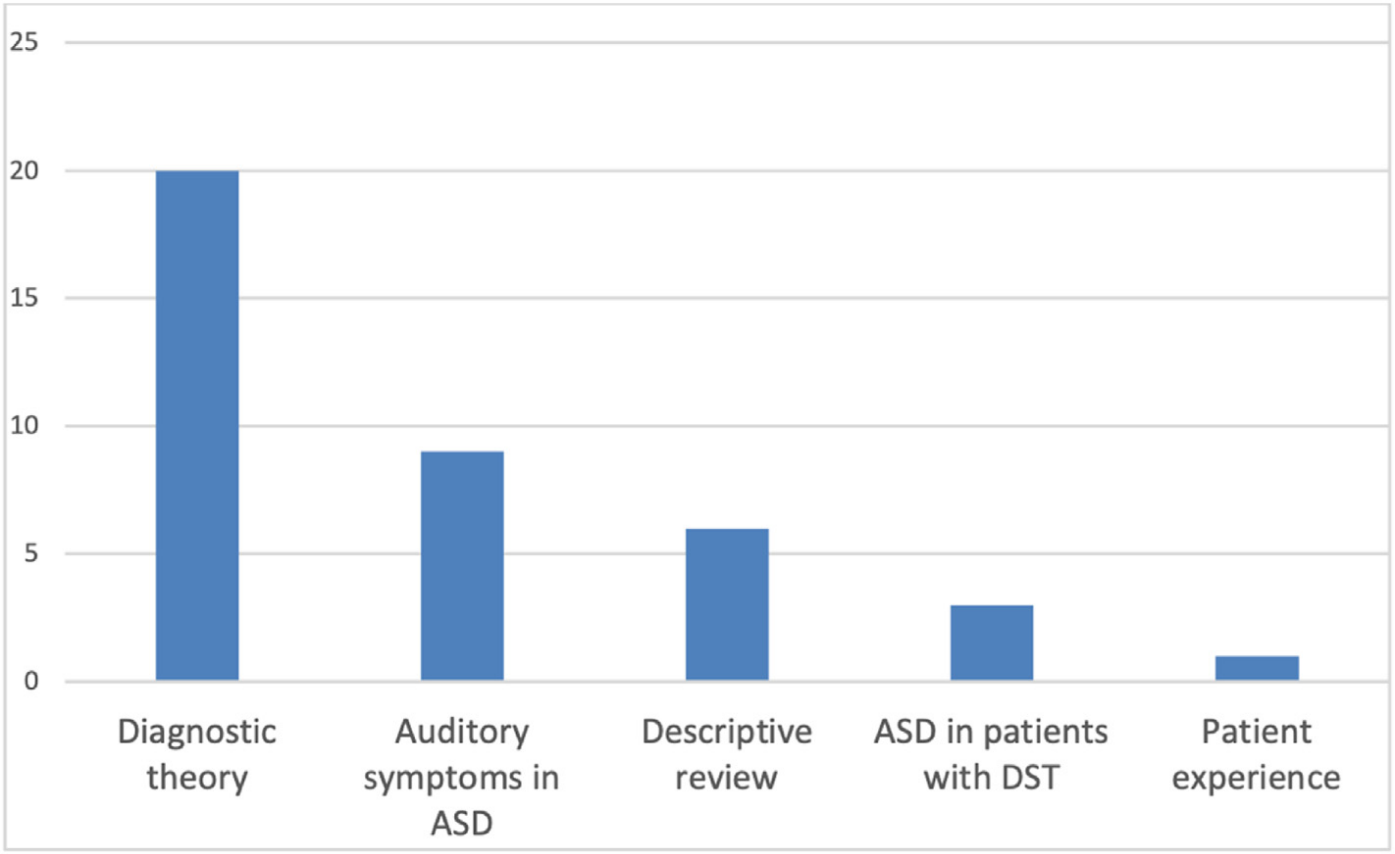
Thematic analysis of the included literature.

The included studies were heterogeneous in study type and outcome measures (Fig. 2). They were therefore classified according to theme (Fig. 3) and these formed the basis of the scoping review. This work involved analysis of published literature only, therefore ethical approval was not required.

## 3 Results and analysis

### 3.1 Study characteristics

Of the 39 included studies, twenty-two were original prospective research, twelve literature reviews, four retrospective case series, and one meta-analysis (Fig. 2). Only five were published in the ENT literature, seventeen originated from neurodevelopmental journals, nine from audiology, five from basic science, two from paediatrics, and one from osteopathic medicine. Twenty of the thirty-nine papers presented diagnostic theory regarding the intersection of ASD and tinnitus and/or DST. Nine papers analysed the prevalence of auditory symptoms including hearing loss in autistic populations, while three papers evaluated the prevalence of ASD among those presenting with auditory symptoms. Six were descriptive reviews and one paper described patient experience. The results of our review are presented according to these five themes.

### 3.2 Diagnostic theory

Twenty studies investigated diagnostic theories for abnormal auditory symptoms in patients with ASD. These explored all levels of the auditory processing pathway.

#### 3.2.1 Inner ear

No studies identified significant inner ear abnormalities in patients with ASD and auditory symptoms. One literature review (Beers et al., 2014) commented on the challenges of behavioural testing in autistic individuals, concluding that there was no evidence of greater levels of hearing loss in autistic children compared to their neurotypical counterparts. A study investigating cochlear microphonic found no difference in children with ASD compared with controls (Osman Dabbous, 2016). Another study found no difference in sound-induced suppression of distortion product otoacoustic emissions in those with and without Asperger's syndrome, a high-functioning form of autism (Kaf and Danesh, 2013). Another study (Ohmura et al., 2019) found that DPOAE suppression was increased in hyperacusis patients, but that this was not well correlated with hyperacusis severity score. A study investigating autistic children with and without auditory hypersensitivity behaviour found superior semicircular canal dehiscence in 29% of subjects with hypersensitivity and in none of those without, though the study numbers were small (Thabet and Zaghloul, 2013).

#### 3.2.2 Brainstem

If inner ear function is largely normal in those with decreased sound tolerance, symptoms may arise from higher processing of auditory stimuli. This process begins in the brainstem, with the cochlear nerve synapsing in the superior olivary nucleus and inferior colliculus. Some evidence of abnormality at this level is indicated by abnormal auditory brainstem responses wave latencies (Dabbous, 2012). Another study found that the physiological suppression of transient evoked otoacoustic emissions by the brainstem-mediated medial olivocochlear reflex was significantly greater in children with ASD and severe hyperacusis, when compared with other children with ASD and neurotypical controls (Wilson et al., 2017). The stapedial reflex, which dampens excessively loud auditory stimuli, is mediated by a short reflex arc involving the auditory brainstem. Ohmura et al. (2019) investigated stapedial reflexes as an index of the level at which a sound is perceived as uncomfortable, finding that the reflex threshold was significantly lower in the autistic group than the control group. The paper concluded that the inner ear is highly sensitive in autistic individuals and that stapedial reflexes could even be used as part of a panel of diagnostic tests for ASD. Another study found stapedial reflexes to be slower, with lower thresholds and greater asymmetry in children with ASD (Lukose et al., 2013). Other evidence of brainstem abnormalities comes from a rat model of autism, created through prenatal thalidomide exposure. Immunostaining demonstrated significant variation in auditory neurone numbers in the superior olivary complex and medial nucleus of the trapezoid body, indicating altered auditory localisation and processing (Ida-Eto et al., 2017; Tsugiyama et al., 2020).

#### 3.2.3 Thalamus

One study explored auditory processing in the thalamus (Mansour et al., 2021) finding smaller nuclei in the auditory region of the thalamus in a rat model of ASD, with fewer connections to ascending nuclei.

#### 3.2.4 Auditory cortex

Two studies examined primary auditory cortex response to sound stimuli, using functional neuroimaging. Responses were found to be slower in autistic children with auditory hypersensitivity compared with children without auditory hypersensitivity and children without ASD (Matsuzaki et al., 2012, 2014). The authors theorize that hypersensitivity may result from a failure of the normal acceleration of the cortical response with age. One study identified normal sensory gating in children with ASD, except those with the lowest IQ scores (Orekhova et al., 2008). Another found little evidence of significant difference between children under five years old with and without autism (Dwyer et al., 2021).

#### 3.2.5 Other

Some researchers argue that DST in ASD is behavioural in aetiology. Lucker (2013) examined 200 children with auditory hypersensitivity, fifty of whom had an ASD diagnosis, and found that most could tolerate sounds above 100 dB, concluding that emotional response, rather than auditory function, causes DST behaviour. A 2010 literature review reached a similar conclusion, arguing that DST in ASD can be explained by fear, and that autistic individuals can learn to alter their behavioural response to distressing sounds (Stiegler and Davis, 2010).

Behavioural responses in rats have been studied using a model of Fragile X syndrome, the most common monogenetic cause of autism. In one study, these rats were found to react faster to auditory stimuli with a wide bandwidth, unlike wild-type rats (Auerbach et al., 2021). These differences were restored to normal when glutamate antagonists were administered, implicating abnormal neurotransmission in the auditory pathway and suggesting potential therapeutic targets for future research.

One paper studied the non-classical pathway of auditory processing, finding that autistic individuals are more likely to perceive a change in the loudness of an auditory stimulus when a concurrent electrical impulse is applied to the wrist (Møller et al., 2005). This gives evidence for interaction between different sensory modalities in autism.

Only one paper considered genetic causes of DST, identifying two genes (CNTN6, CNTN5) that are expressed at high levels in the auditory pathway, and were found to have mutations in some autistic subjects at a significantly higher rate than in the general population (Mercati et al., 2017).

### 3.3 Descriptive reviews

A Cochrane review (Sinha et al., 2011) summarised six randomised controlled trials of auditory integration therapy in ASD, concluding no evidence for its effectiveness, though limited by disparate outcome measures. A systematic review focusing on terminology (Stefanelli et al., 2020) concluded that disparity in terminology renders synthesis of literature challenging. Gomes et al. (2008) noted a gap in the literature, recommending further research to better understand communicative behaviours in ASD. Moller (Møller, 2009) proposed that autism and tinnitus are both caused by alterations in neural plasticity. McCullagh et al. (2020) reviewed auditory processing deficits in Fragile X syndrome. Smith et al. (2019) proposed the utility of auditory testing to screen for.

ASD.

### 3.4 ASD in patients presenting with DST

Two papers were paediatric hyperacusis case series. Myne (Myne and Kennedy, 2018) observed that 13% of 61 children referred with hyperacusis had a pre-existing ASD diagnosis. Amir (Amir et al., 2018) observed that of 412 children referred with hyperacusis, 60% had a pre-existing ASD diagnosis, and 99% had normal audiometry. An epidemiological study performed in Finland surveyed 4397 eight-year-olds and found that auditory hypersensitivity indicated a 22-fold increase in prevalence of ASD (Jussila et al., 2020).

### 3.5 Auditory symptoms in ASD

The Finnish epidemiological study reported auditory hypersensitivity in 43% of children with ASD, and in 3% of children without ASD (Jussila et al., 2020). The only meta-analysis included in our scoping review gives a prevalence of 40% for hyperacusis in those with ASD, and a lifetime prevalence of 60%, increasing with age (Williams et al., 2021a). In a survey of 55 children and adults with highfunctioning autism, 69% reported hyperacusis (Danesh et al., 2015). Williams’ 2020 literature review quotes a prevalence of 38–45% for DST in autistic individuals (Williams et al., 2021b). Another 2021 review quotes similar figures (Danesh et al., 2021).

### 3.6 Patient experience

One study explored patient and parental experience of DST in autistic children by means of a questionnaire. Families described frequent negative reaction to sounds, including reacting to the anticipation of sound. Loud, high-pitched, and sudden sounds were rated the most distressing, and management strategies were described including pre-warning the child about a sound, avoiding noisy places and taking ‘time out’ where necessary. Many parents commented that aversion to sounds prevented their child from participating in social.

Activities (Scheerer et al., 2022).

## 4 Discussion

This is the first literature review on this theme from an ENT perspective, and therefore has significant potential to influence future research. It draws on publications from multidisciplinary areas, particularly the neurodevelopmental literature, highlighting auditory symptoms in ASD as an intersectional theme which has relevance across several specialties. Its clinical applicability is of great importance considering that undiagnosed ASD may present to ENT and Audiology in the form of unexplained auditory symptoms. The concept of considering a potential undiagnosed condition is an inherent limitation of this work.

Our primary aim was to review the literature on ASD and auditory symptoms, exploring the intersection of these themes and identifying evidence for auditory symptoms as a presenting feature of ASD in ENT and Audiology services. The literature is heterogeneous, and several themes emerge. Our review analyses literature investigating diagnostic theories for the correlation between ASD and auditory symptoms, exploring the entire auditory pathway, including emotional and behavioural responses to sound. There have been several promising discoveries, and the pathophysiological mechanism of tinnitus and DST in individuals with DST is likely to be multifactorial. Literature reviews have highlighted disparities in terminology, gaps in the literature, proposals for future research on communicative behaviours and the potential for auditory testing to screen for ASD, and that there is limited evidence for the effectiveness of auditory integration therapy. An interesting feature of published research in the neurodevelopmental literature is that tinnitus and DST are rarely defined in terms of their laterality or specific nature.

Evidence for auditory symptoms as a presenting feature of ASD arises exclusively from paediatric populations in the existing literature; there is currently no published data on the incidence of ASD amongst adults presenting with tinnitus and DST. There is however a growing body of evidence highlighting a very high incidence of tinnitus and DST in both children and adults with an ASD diagnosis, indicating a significant potential that undiagnosed ASD may be presenting to ENT and Audiology services in the form of tinnitus and DST.

But what are the implications of this? Our review highlights a need for focussed clinical research, and a potential learning need for professionals who investigate and manage patients with tinnitus and DST. Screening for a lifelong neurodevelopmental condition in a population presenting with an apparently unrelated symptom, carries implications for both patients and clinical services. Screening for ASD has been previously explored only in

Psychiatry outpatient populations. The concept of diagnosing ASD in ENT and Audiology services introduces numerous factors which need to be considered, including appropriate communication and patient counselling regarding the impact of such a diagnosis for patients, appropriate training of staff, and the subsequent impact of a new ASD diagnosis for primary care and other services. It is likely that a multidisciplinary approach would be beneficial in providing a holistic clinical service with robust governance.

A secondary aim of this review was to outline a research agenda based on themes emerging. Based on our in-depth analysis of existing literature and our observations of topics which could usefully be explored to enhance our clinical understanding of this theme, we recommend the following:

a) Collaborative research between disciplines, to optimise the multidisciplinary
  approach.
b) Robust, ethical screening studies in both paediatric and adult populations, to identify the incidence of diagnosed and undiagnosed ASD presenting to ENT and Audiology
  services with tinnitus and/or DST.
c) Formulation of a core outcome set for future trials involving auditory symptoms in individuals with ASD, in order that these can be compared and summarised.
d) Clarification of terminologies used to describe and investigate auditory symptoms in
  patients with ASD, via a consensus process.
e) Studies to ascertain whether individuals with ASD are likely to experience unilateral and/or bilateral tinnitus and DST, and the nature of these, to refine diagnostic
  algorithms.
f) Qualitative research to better understand the experience and impact of auditory
  symptoms for individuals with ASD.
g) Trials of therapies for auditory symptoms in individuals with ASD.
h) Further research to clarify and understand the physiological mechanisms of auditory
  symptoms in ASD, which are likely to be multifactorial.
i) Engagement of autistic researchers, autistic advocates, and robust patient and public involvement in any future work which affects or implicates autistic populations.

## 5 Conclusion

There is a growing body of literature demonstrating a very high incidence of tinnitus and DST in individuals with ASD. This suggests a high possibility that patients presenting to ENT and Audiology services with these symptoms may have a primary diagnosis of ASD to account for their otherwise unexplained auditory symptoms.

This also indicates a learning need for professionals who investigate and manage tinnitus and DST, as well as a requirement to refine existing protocols, which currently focus on the identification and exclusion of identifiable inner ear conditions, to consider neurodevelopmental disorders as a potential primary diagnosis. A collaborative, multidisciplinary model is essential, considering the potential implications of a new ASD diagnosis.

There is a pressing need for further research on this clinically important theme. We outline a research agenda with a focus on multidisciplinary collaboration, stakeholder engagement, clinical screening, and clarification of pathophysiological mechanisms and terminology.

## Declaration of funding sources

This research did not receive any specific grant from funding agencies in the public, commercial or not-for-profit sectors.

## Ethics approval

• This was a literature review and therefore ethical approval was not required.

## Data sharing and availability

• No primary data were used in the production of this manuscript.
• Our Search Strategy is available in Table 1.
• This study and its analysis were not pre-registered.

## Authorship statement

ES designed the work; ST, SL, JB, ES acquired and analysed data, drafted, revised, and approved the manuscript; ST and ES agree to be accountable for all aspects of the work. All authors have read and approved this manuscript.

## Declaration of competing interest

• The authors have no relevant financial or non-financial interests to disclose.
